# Clinical use and adjustment of ultrasound elastography for breast lesions followed WFUMB guidelines and recommendations in the real world

**DOI:** 10.3389/fonc.2022.1022917

**Published:** 2022-11-24

**Authors:** Lei Tang, Yuqun Wang, Pingping Chen, Man Chen, Lixin Jiang

**Affiliations:** ^1^ Department of Ultrasound, Renji Hospital, School of Medicine, Shanghai Jiao Tong University, Shanghai, China; ^2^ Department of Ultrasound Medicine, Tongren Hospital, School of Medicine, Shanghai Jiao Tong University, Shanghai, China

**Keywords:** strain elastography, shear wave elastography, breast cancer, BI-RADS, biopsy

## Abstract

**Objective:**

This study aimed to explore the value of strain elastography (SE) and shear wave elastography (SWE) following the World Federation of Ultrasound in Medicine and Biology (WFUMB) guidelines and recommendations in the real world in distinguishing benign and malignant breast lesions and reducing biopsy of BI-RADS (Breast Imaging Reporting and Data System) 4a lesions.

**Methods:**

This prospective study included 274 breast lesions. The elastography score (ES) by the Tsukuba score, the strain ratio (SR) for SE, and Emax for SWE of the lesion(A) and the regions(A’) included the lesion and the margin (0.5-5 mm) surrounding the lesion were measured. The sensitivity, specificity, and AUC were calculated and compared by the cutoff values recommended by WFUMB guidelines.

**Results:**

When scores of 1 to 3 were classified as probably benign by WFUMB recommendation, the ES was significantly higher in malignant lesions compared to benign lesions (p < 0.05) in all lesions. For the cohort by size >20 mm, the sensitivity was 100%, and the specificity was 45.5%. ES had the highest AUC: 0.79(95% CI 0.72-0.86) with a sensitivity of 96.2%, and a specificity of 61.8% for the cohort by size ≤20 mm. For the Emax-A’-S2.5mm, when the high stiffness would be considered with Emax above 80 kPa in SWE, the malignant lesions were diagnosed with a sensitivity of 95.8%, a specificity of 43.3% for all lesions, a sensitivity of 88.5% for lesions with size ≤20 mm, and sensitivity of 100.0% for lesions with size >20 mm. In 84 lesions of BI-RADS category 4a, if category 4a lesions with ES of 1-3 points or Emax-A’-S2.5 less than 80 kPa could be downgraded to category 3, 52 (61.9%) lesions could be no biopsy, including two malignancies. If category 4a lesions with ES of 1-3 points and Emax-A’-S2.5 less than 80kPa could be downgraded to category 3, 23 (27.4%) lesions could be no biopsy, with no malignancy.

**Conclusions:**

The elastography score for SE and Emax-A’ for SWE after our modification were beneficial in the diagnosis of breast cancer. The combination of SWE and SE could effectively reduce the biopsy rate of BI-RADS category 4a lesions.

## Introduction

Breast cancer is the most common malignant tumor among females, with an incidence of up to 30% (1). Although mammography was a valuable tool for screening for breast cancer in clinical practice, the role of ultrasound in the diagnosis of breast cancer was gradually being widely recognized (2, 3). Ultrasound breast cancer detection was very similar to mammography and could be used as a supplement to mammography (4). It was also an important screening tool for younger women and women with dense breasts (2). The Breast Imaging Reporting and Data System (BI-RADS) of the American College of Radiology (ACR) is widely used in most countries (5–9). The fifth edition of BI-RADS (2013) has been revised based on accumulated clinical practice, and the elastic part has been added (10). However, it contained only part of the strain elastography and no objective reference value was used (11).

Elastography could assist in diagnosis and differentiation according to the stiffness difference between tumor area and surrounding tissue as well as the stiffness difference between benign and malignant lesions, which had brought clinical benefits. Two main modes of elastography have become established in clinical practice: strain elastography (SE) and shear wave elastography (SWE) (12). SE and SWE have shown significant value in previous studies (13–16). SWE could provide useful diagnostic information for tissue stiffness to improve the accuracy of B-ultrasound diagnosis. However, the optimal application of SWE and strain elastography in clinical breast imaging is still under investigation (17).

In 2015, the breast section of the Guidelines and Recommendations for Elastography produced under the auspices of the World Federation of Ultrasound in Medicine and Biology (WFUMB) was published (18). It was believed that the two elastic imaging modes had similar diagnostic capabilities and the diagnostic parameters were recommended. ​Nonetheless, the effect of applying this standard in the practical application has not been determined. Since the role of the “Stiff Rim” sign obtained by shear wave elastography was considered helpful in the diagnosis of breast cancer, recent studies have shown that stiffness information around the mass (shell) was significant assistance (19–21). The diagnosis of breast cancer was more and more standardized by BI-RADS, but a large number of benign lesions were biopsied, which brought significant anxiety and physical and mental harm to patients. The diagnostic specificity could be increased by elastography, but further research was needed to reduce unnecessary biopsies (13, 17, 22).

This study aimed to verify the diagnostic ability of two elastic ultrasound methods in breast lesions of different sizes by evaluating the clinical application of the elasticity indexes recommended by WFUMB, and explore whether the supplement of edge information could further enhance the differential ability of benign and malignant masses, and provide the diagnostic basis for reducing unnecessary biopsy of category 4a.

## Materials and methods

### Patients

This study was approved by our hospital ethics committee. Written informed consent was obtained from all patients participating in the study.

From July 2019 to April 2021, a total of 297 consecutive patients with breast lesions were enrolled in this study. The inclusion criteria were as follows (1): breast lesions were visible on the conventional US (2); no biopsy was performed before US examination (3); patients underwent the preoperative US and SWE examination and the breast surgery in our hospital in a week and (4) one lesion with the highest BI-RADS category or with the largest diameter in the same BI-RADS category was selected in each patient. Twenty-three patients were excluded because of the following reasons (1): male patients (n =5) (2); SWE examination was not performed (n =6) (3); simple cyst or simple ductal dilation on the conventional US (n =3) (4); lack of normal breast tissues surrounding the enormous lesions (large than 5cm) for the elastic image (n =6) (5); lesions with NAC treatments before surgery (n =2) and (6) no final histological results (n =1). Finally, 274 lesions were divided into two cohorts based on the diameter of ≤2cm or >2cm on the ultrasound images. Because in the AJCC tumor-node-metastasis (TNM) staging system, the boundary between the T1 stage and T2 stage of breast cancer is 2cm. We hoped that the size of the lesion should match the pathological stage as much as possible. However, due to the relative lag of pathological acquisition, we hoped to make the corresponding prediction before surgery, so the maximum diameter seen by ultrasound was taken as the classification standard.

### US and elastography evaluation

The conventional US and elastography were performed using a Resona 7 diagnostic US system (Mindray Medical International, Shenzhen, China) with a linear-array transducer (L14–5 MHz). The conventional US examination and a series of elastography for the lesions were performed by a specialist with at least 15 years of experience in breast ultrasound.

The patients were in a supine position and breathing smoothly in a quiet environment during the breast ultrasound. The conventional US was performed to determine the target lesion. The lesions were classified by the fifth edition of the Breast Imaging Reporting and Data System (BI-RADS). The highest BI-RADS category lesion or the largest lesion in the same category was designated as the target lesion. Only one target lesion was included in each patient.

SE and SWE were performed based on WFUMB guidelines by the same radiologist (18). The probe was kept perpendicular to the skin and lightly touched the skin with minimum manual compression. Use the quality control chart of the instrument to ensure that the image quality was stable. When five stars appeared in SE and uniform green appeared in SWE, the image quality was considered higher. Conventional ultrasound and elastography images were displayed on the left and right sides of the same image. The maximum section of the lesion was selected as the elastic region.

The elastography images were analyzed by a single radiologist with 5 years of experience in breast ultrasound, who was blinded to the conventional US and histopathological diagnosis results. Before this study, the radiologist with 5 years of experience was systematically trained in the analysis of elastography, and the Kappa consistence for the diagnostic agreement could reach above 0.75, contrasted with the radiologist with 15 years of experience. The ROI was plotted using an ellipse based on the lesion area to ensure that all lesions could be covered. A five-point scale for SE, called elastography score(ES), was visually graded by the Tsukuba score for the stiffness of the lesion (23). The strain ratio (SR) for SE was calculated by the fat-lesion ratio (LFR), which was the target lesion compared to subcutaneous fat. Emax, quantitative elastographic features of the stiffness of the lesion(A) and the regions(A’), which included the lesion and the margin (0.5-5 mm) surrounding the lesion in 0.5mm increments, were measured using the shell function according to shell size in SWE. The elastic maximum of the shell size ‘n’ of the regions(A’) was defined as Emax-A’-Sn.

### Statistical analysis

The chi-square and Fisher exact tests were used for categorical variables and the independent samples t-test was used for continuous variables in the different cohorts. The area under the curve (AUC) of the receiver operating characteristic curve was used for parameter selection and assessment. The optimum cutoff value was determined through the Youden index (maximum of sensitivity + specificity - 1). Specificity, sensitivity, and AUC (95% confidence interval [CI]) were an estimate of diagnostic accuracy. Statistical analyses were performed using SPSS 23.0 software, with a value of *P*<0.05 being considered significant.

The diagnostic value of ES, SR, and Emax was evaluated and compared by different cutoff values in differentiating benign and malignant breast masses. The diagnostic value of the combination of ES and/or Emax in reducing the biopsy of BI-RADS 4a lesions was analyzed.

## Results

### Study population

The patients ranged in age from 17 to 88 years, with the average age being 45.9 ± 15.5 years. The lesions ranged in size from 3.5 to 47.1 mm, with the average lesion size being 16.9 ± 9.1 mm. Among the 274 lesions (71 malignant, 203 benign), 196 lesions (26 malignant, 170 benign) were size ≤20 mm and 78 lesions (45 malignant, 33 benign) were size >20 mm, as shown in **Table 1**.

**Table 1:** The characteristics of patients and breast lesions.

Characteristics	All lesions	Malignant	Benign	*P*	Cohort by size ≤20 mm	Malignant	Benign	*P*	Cohort by size >20 mm	Malignant	Benign	*P*
Mean age, years	45.9 ± 15.5	58.9 ± 13.4	41.3 ± 13.5	0.000	44.5 ± 14.8	59.6 ± 14.6	42.2 ± 13.5	0.000	49.4 ± 16.7	58.6 ± 12.9	36.9 ± 12.6	0.000
Mean tumor size, mm	16.9 ± 9.1	24.0 ± 9.6	14.4 ± 7.5	0.000	12.1 ± 3.9	14.6 ± 4.3	11.8 ± 3.7	0.001	28.9 ± 7.1	29.5 ± 7.4	28.1 ± 6.8	0.408
0-10mm	56	5	60									
10-20mm	131	21	110									
20-30mm	51	29	22									
30-40mm	20	10	10									
40-50mm	7	6	1									
Case numbers	274	71	203		196	26	170		78	45	33	
Category 3	119	0	119		102	0	102		17	0	17	
Category 4a	84	7	77		70	7	63		14	0	14	
Category 4b	18	12	6		12	7	5		6	5	1	
Category 4c	21	20	1		7	7	0		14	13	1	
Category 5	32	32	0		5	5	0		27	27	0	

BI-RADS, Breast Imaging Reporting and Data System.

From pathology diagnosis, the malignant lesions included invasive ductal carcinoma (n =46), ductal carcinoma *in situ* (n =13), solid papillary carcinoma (n =5), mucinous carcinoma (n =4), invasive lobular carcinoma (n =1), medullary carcinoma(n=1), and secretory carcinoma(n=1). The benign lesions included fibroadenoma (n =135), adenosis (n =38), intraductal papilloma (n =15), inflammation (n =11), and fibroadenomatous hyperplasia (n =4).

BI-RADS classification was shown in **Table 1**, and there were statistical differences between benign and malignant lesions in three cohorts (*P* =0.000).

### Three kinds of elastography parameters in different cohorts

For ES, no malignant lesion was scored 2 points, and almost all malignant lesions but one were scored 4-5 points in this study. There was a significant statistical difference between benign and malignant lesions. The strain elastic parameters (ES, SR) and SWE Emax-A were statistically different in the cohorts regardless of whether the lesion was larger than 2cm or not (**Table 2**).

**Table 2:** The elastography characteristics of breast lesions.

Characteristics	All lesions	Malignant	Benign	*P*	Cohort by size ≤20 mm	Malignant	Benign	*P*	Cohort by size >20 mm	Malignant	Benign	*P*
ES				0.000				0.000				0.000
ES 2	10	0	10		9	0	9		1	0	1	
ES 3	111	1	110		97	1	96		14	0	14	
ES 4	91	18	73		67	9	58		24	9	15	
ES 5	62	52	10		23	16	7		39	36	3	
SR	3.7 ± 1.5	4.9 ± 1.9	3.3 ± 1.2	0.000	3.6 ± 1.4	5.1 ± 2.1	3.3 ± 1.1	0.000	4.2 ± 1.7	4.8 ± 1.8	3.4 ± 1.3	0.000
Emax-A, kPa	85.4 ± 38.1	110.2 ± 44.3	76.7 ± 31.4	0.000	75.2 ± 29.9	92.7 ± 30.9	72.5 ± 29.0	0.001	111.0 ± 44.1	120.3 ± 47.9	98.4 ± 35.0	0.029
Emax-A’-S2.5mm, kPa	102.7 ± 42.0	129.7 ± 44.4	93.3 ± 36.8	0.000	93.2 ± 37.5	120.6 ± 44.0	89.0 ± 34.6	0.000	126.7 ± 43.4	134.9 ± 44.3	115.4 ± 40.2	0.05

ES, elastography score; SR, strain ratio.

When scores of 1 to 3 were classified as probably benign by WFUMB recommendation, the ES was significantly higher in malignant lesions compared to benign lesions (*P* < 0.05) in all lesions. For the cohort by size >20 mm, the sensitivity was 100%, and the specificity was 45.5%. ES had the highest AUC: 0.79(95% CI 0.72-0.86) with a sensitivity of 96.2%, and a specificity of 61.8% for the cohort by size ≤20 mm. When the score of 5 was classified as probably malignant, ES had the highest AUC: 0.86(95% CI 0.77-0.94) with a sensitivity of 80.0%, a specificity of 90.9% for the cohort by size >20 mm, and the highest specificity of 95.9% for the cohort by size ≤20 mm (**Table 3**).

**Table 3:** The diagnostic ability of elastic parameters in breast masses of different sizes.

		Cut-off value	*P*	Sensitivity	Specificity	AUC (95%CI)
ES						
	All lesion	3-4*	0.000	98.6%	59.1%	0.79 (0.74-0.84)
		4-5	0.000	73.2%	95.1%	0.84 (0.78-0.91)
	Size ≤20 mm	3-4*	0.000	96.2%	61.8%	0.79 (0.72-0.86)
		4-5	0.000	61.5%	95.9%	0.79 (0.67-0.90)
	Size >20 mm	3-4*	0.000	100%	45.5%	0.73 (0.61-0.85)
		4-5	0.000	80.0%	90.9%	0.86 (0.77-0.94)
SR						
	All lesion	5.345	0.000	40.8%	95.6%	0.68 (0.60-0.76)
		4.5*	0.000	50.7%	81.8%	0.66 (0.59-0.74)
	Size ≤20 mm	5.345	0.000	46.2%	96.5%	0.71 (0.59-0.84)
		4.860	0.000	53.8%	90.6%	0.72 (0.60-0.84)
		4.5*	0.000	57.7%	83.5%	0.71 (0.59-0.83)
	Size >20 mm	5.345	0.004	37.8%	90.9%	0.64 (0.52-0.77)
		3.905	0.003	64.4%	69.7%	0.67 (0.55-0.79)
		4.5*	0.082	46.7%	72.7%	0.60 (0.47-0.72)
Emax-A						
	All lesions	82.295	0.000	80.3%	65.5%	0.73 (0.66-0.80)
		80*	0.000	80.3%	63.5%	0.72 (0.65-0.79)
		60*	0.000	95.8%	31.0%	0.63 (0.57-0.70)
	Size ≤20 mm	82.295	0.000	69.2%	71.2%	0.70 (0.59-0.81)
		80*	0.000	69.2%	69.4%	0.69 (0.58-0.80)
		60*	0.005	92.3%	35.3%	0.64 (0.54-0.74)
	Size >20 mm	96.370	0.001	71.1%	66.7%	0.69 (0.57-0.81)
		82.295	0.017	86.7%	36.4%	0.62 (0.49-0.74)
		80*	0.035	86.7%	33.3%	0.60 (0.47-0.73)
		60*	0.401	97.8%	9.1%	0.53 (0.40-0.67)
Emax-A’-S2.5mm						
	All lesions	90.3	0.000	88.7%	58.1%	0.73 (0.67-0.80)
		80*	0.000	95.8%	43.3%	0.70 (0.63-0.76)
		60*	0.002	98.6%	15.3%	0.57 (0.50-0.64)
	Size ≤20 mm	106.7	0.000	65.4%	77.1%	0.71 (0.60-0.83)
		90.3	0.000	76.9%	62.9%	0.70 (0.60-0.80)
		80*	0.001	88.5%	47.1%	0.68 (0.58-0.78)
		60*	0.147	96.2%	17.1%	0.57 (0.46-0.68)
	Size >20 mm	90.3	0.001	95.6%	33.3%	0.64 (0.52-0.77)
		80*	0.002	100.0%	24.2%	0.62 (0.49-0.75)
		60*	0.176	100.0%	6.1%	0.53 (0.40-0.67)

AUC, area under the curve; CI, confidence interval.

* The cutoff values were recommended by WFUMB guidelines.

For the cohort by size ≤20 mm, SR had the highest specificity of 83.5% by cut-off value of 4.5 according to WFUMB recommendation, the highest specificity of 96.5% by cut-off value of 5.345 (**Table 3**).

When the high stiffness would be considered with Emax above 80 kPa in SWE by WFUMB recommendation, the malignant lesions were diagnosed with a sensitivity of 80.3%, a specificity of 63.5% for all lesions. The poor results were shown in the cohorts by size ≤20 mm and by size >20 mm. For the cohort by size ≤20 mm, the sensitivity was 69.2%, and the specificity was 69.4%. For the cohort by size >20 mm, the sensitivity was 86.7%, and the specificity was 33.3% (**Table 3**).

### Diagnostic performance of the quantitative SWE features of the lesion and shell (E-A’)

In all lesions, it was measured for the Emax for the lesion(A) and shell(A’) with the different width shell (0.5-5mm), and the values of Emax-A’ were significant statistical differences between benign and malignant lesions (*P* =0.000) (**Table 4**). The Emax-A’-S2.5mm had the highest AUC: 0.77(95% CI 0.71-0.83) with a sensitivity of 88.7%, and a specificity of 58.1%.

**Table 4:** Diagnostic ability of E Max in mass regions with different width shells in all lesions.

	Pathology	Mean	t	*P*	AUC (95%CI)
Emax-A	Benign	76.7 ± 31.4	-5.867	0.000	0.77 (0.71-0.83)
	Malignant	110.2 ± 44.3			
Emax-A’-S5mm	Benign	103.6 ± 37.4	-5.968	0.000	0.73 (0.67-0.79)
	Malignant	136.1 ± 45.2			
Emax-A’-S4.5mm	Benign	102.0 ± 37.4	-6.087	0.000	0.73 (0.67-0.80)
	Malignant	135.3 ± 45.4			
Emax-A’-S4mm	Benign	100.2 ± 37.3	-6.028	0.000	0.73 (0.67-0.80)
	Malignant	133.2 ± 45.8			
Emax-A’-S3.5mm	Benign	97.6 ± 37.5	-6.363	0.000	0.75 (0.69-0.81)
	Malignant	132.7 ± 46.3			
Emax-A’-S3mm	Benign	95.3 ± 37.2	-6.677	0.000	0.76 (0.70-0.82)
	Malignant	131.8 ± 46.2			
Emax-A’-S2.5mm	Benign	93.3 ± 36.8	-6.790	0.000	0.77 (0.71-0.83)
	Malignant	129.7 ± 44.4			
Emax-A’-S2mm	Benign	91.1 ± 35.7	-6.717	0.000	0.77 (0.71-0.83)
	Malignant	126.0 ± 43.1			
Emax-A’-S1.5mm	Benign	88.4 ± 34.9	-6.405	0.000	0.76 (0.70-0.82)
	Malignant	121.0 ± 42.1			
Emax-A’-S1mm	Benign	84.0 ± 33.5	-6.511	0.000	0.76 (0.70-0.82)
	Malignant	116.2 ± 42.0			
Emax-A’-S0.5mm	Benign	80.7 ± 32.5	-6.536	0.000	0.76 (0.70-0.82)
	Malignant	112.5 ± 42.4			

AUC, area under the characteristic curve; CI, confidence interval.

For the Emax-A’-S2.5mm, when the high stiffness would be considered with Emax above 80 kPa in SWE, the malignant lesions were diagnosed with a sensitivity of 95.8%, a specificity of 43.3% for all lesions, a sensitivity of 88.5% for lesions with size ≤20 mm, and a sensitivity of 100.0% for lesions with size >20 mm (**Table 3**).

### The combined diagnosis of SE and SWE

When the lesions with ES of 4-5 points and Emax-A’-S2.5 more than 80kPa, the malignant lesions were diagnosed with a sensitivity of 94.4%, a specificity of 70.0% for all lesions, a sensitivity of 84.6% for lesions with size ≤20 mm, and a sensitivity of 100.0% for lesions with size >20 mm. When the lesions with ES of 4-5 points or Emax-A’-S2.5 were more than 80kPa, the malignant lesions were diagnosed with a sensitivity of 100.0% for all cohorts (**Table 5**).

**Table 5:** The combined diagnostic capability of ES and Emax-A’-S2.5mm.

		χ^2^	*P*	Sensitivity	Specificity	AUC (95%CI)
ES≥4 AND Emax-A’-S2.5mm>80kPa						
	All lesion	87.416	0.000	94.4%	70.0%	0.82 (0.77-0.87)
	Size ≤20 mm	35.803	0.000	84.6%	74.7%	0.80 (0.71-0.89)
	Size >20 mm	25.325	0.000	100.0%	45.5%	0.73 (0.61-0.85)
ES≥4 OR Emax-A’-S2.5mm>80kPa						
	All lesion	30.408	0.000	100.0%	32.5%	0.68 (0.60-0.76)
	Size ≤20 mm	12.599	0.000	100.0%	34.1%	0.67 (0.58-0.76)
	Size >20 mm	9.665	0.002	100.0%	24.2%	0.62 (0.49-0.75)

AUC, area under the characteristic curve; CI, confidence interval.

### The value of ultrasound elastography in BI−RADS category 4a lesions

**Figure 1:**
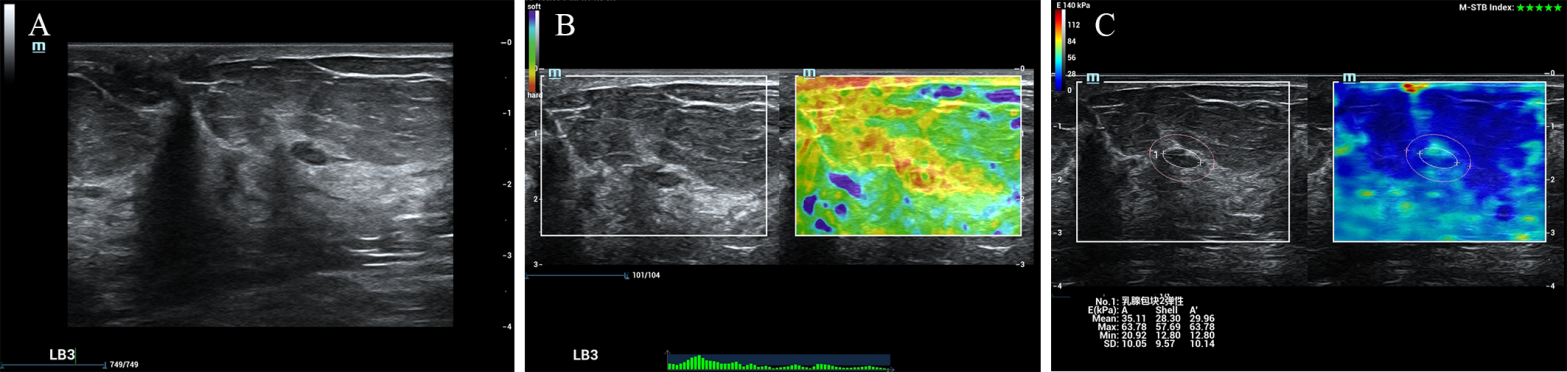
The breast cancer was downgraded by the Emax-A’-S2.5 below 80kpa. A 65-year-old female patient had a lesion on her left breast. Pathologically confirmed intraductal solid papillary carcinoma. The size of the lesion was 6.6mm and ultrasound images of the breast lesion were evaluated as BI-RADS 4a **(A)**, with an elasticity score of 5 **(B)**, Emax-A of 63.8kpa and Emax-A’-S2.5 of 63.8kpa **(C)**.

**Figure 2:**
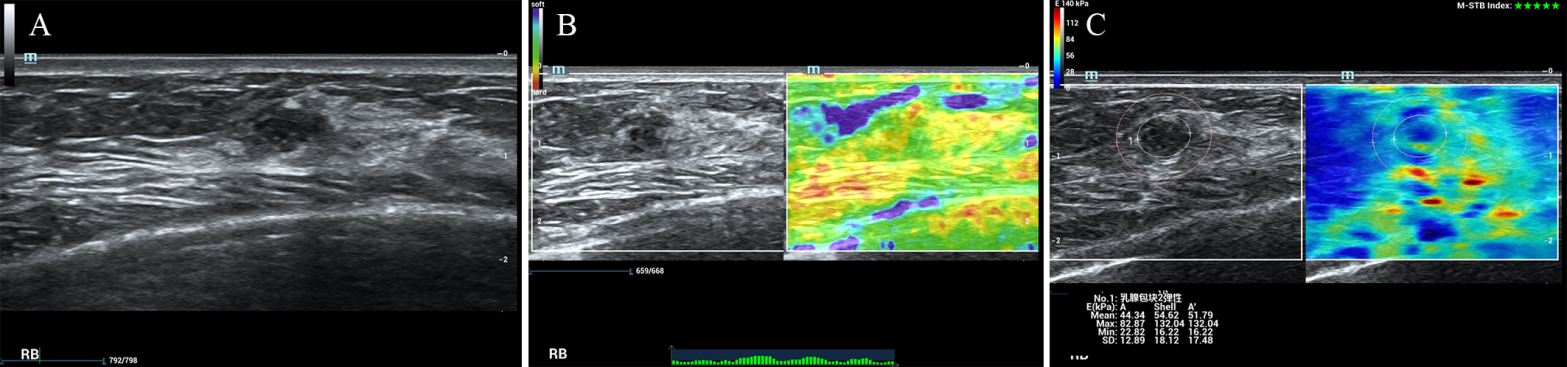
The breast cancer was downgraded by the ES of 1-3 points. A 47-year-old female patient had a lesion on her right breast. Pathologically confirmed middle-grade ductal carcinoma in situ. The size of the lesion was 9mm and ultrasound images of the breast lesion were evaluated as BI-RADS 4a **(A)**, with an elasticity score of 3 **(B)**, Emax-A of 82.9kpa and Emax-A’-S2.5 of 132.0kpa **(C)**.

**Figure 3:**
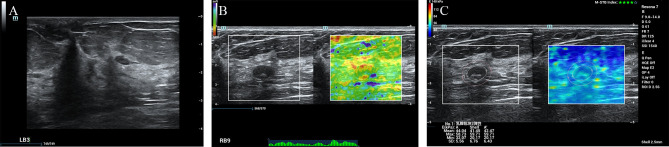
The fibroadenoma was downgraded by the ES of 1-3 points and the Emax-A’-S2.5 below 80kpa. A 67-year-old female patient had a lesion on her right breast. Pathologically confirmed fibroadenoma. The size of the lesion was 11.9mm and ultrasound images of the breast lesion were evaluated as BI-RADS 4a **(A)**, with an elasticity score of 2 **(B)**, Emax-A of 58.7kpa and Emax-A’-S2.5 of 59.71kpa **(C)**.

According to the ROC analysis, the ES with scores of 4 to 5 and E Max for A’ with shell 2.5mm above 80 kPa in SWE had higher sensitivity for breast cancer diagnosis. In this study, there were 84 lesions of BI-RADS category 4a. For Emax-A’-S2.5 below 80kpa, 34(40.5%) category 4a lesions (33 benign lesions and one malignancy) could be downgraded to category 3. The malignant lesion was intraductal solid papillary carcinoma (Emax-A’-S2.5 = 63.8kpa, size=6.6mm) (**Figure 1**). For ES of 1-3 points, 40 (47.6%) category 4a lesions (39 benign lesions and one malignancy) could be downgraded to category 3. The malignant lesion was middle-grade ductal carcinoma *in situ* (ES=3 points, size=9mm) (**Figure 2**). If category 4a lesions with ES of 1-3 points or Emax-A’-S2.5 less than 80kPa could be downgraded to category 3, 52 (61.9%) lesions could be no biopsy, including two malignancies. If category 4a lesions with ES of 1-3 points and Emax-A’-S2.5 less than 80kPa could be downgraded to category 3, 23 (27.4%) lesions could be no biopsy, with no malignancy (**Figure 3**).

### The value of ultrasound elastography in breast cancer

71 cases of breast cancer were classified according to the TNM staging system (**Table 6**). There was 1 lesion (T0N0M0) with ES 3 score, 14 lesions with Emax-A ≤80 kPa, and only 3 cases (T1N0M0) with Emax-A’-S2.5mm ≤ 80kPa. If a biopsy was recommended at an ES of 4-5 points or an Emax-a’-S2.5 greater than 80 kPa for a lesion, no malignant lesion would be missed.

**Table 6:** BI-RADS and elastography characteristics of breast cancer follow TNM stage.

	BI-RADS				ES			ES ≤3	ES≥4	SR ≤ 4.5	SR>4.5	Emax-A ≤80 kPa	Emax-A>80 kPa	Emax-A’-S2.5mm ≤ 80kPa	Emax-A’-S2.5mm>80kPa	All
	4a	4b	4c	5	3	4	5									
T0N0M0	4	1	3	1	1	6	2	1	8	7	2	2	7	0	9	9
T1N0M0	2	4	4	6	0	2	14	0	16	7	9	7	9	3	13	16
T1N1M0	0	1	2	2	0	1	4	0	5	1	4	1	4	0	5	5
T1N2M0	0	1	1	1	0	1	2	0	3	2	1	1	2	0	3	3
T2N0M0	1	5	1	9	0	5	11	0	16	9	7	2	14	0	16	16
T2N1M0	0	0	7	5	0	1	11	0	12	4	8	1	11	0	12	12
T2N2M0	0	0	2	7	0	2	7	0	9	5	4	0	9	0	9	9
T4N2M0	0	0	0	1	0	0	1	0	1	0	1	0	1	0	1	1
All	7	12	20	32	1	18	52	1	70	35	36	14	57	3	68	71

BI-RADS, Breast Imaging Reporting and Data System; TNM, tumor-node-metastasis; ES, elastography score; SR, strain ratio.

## Discussion

This study explored the actual application of guidelines and recommendations for elastography by WFUMB in the diagnosis of breast lesions. It was compared for the diagnostic capabilities of different parameters in different cohorts, based on the diameter of ≤2cm or >​2cm. The recommended diagnostic criteria of SWE were explored to improve by extending the boundary of the lesions. In addition, the combination of ES or/and Emax-A’-S2.5 could significantly reduce the biopsy rate of BI-RADS category 4a lesions.

The performance of ES was surprising among the recommended elastic parameters of the strain formula. The sensitivity of 4 to 5 scores for the diagnosis of malignant lesions was 98.6%, especially the sensitivity was as high as 100% for the sizes of lesions than 2 cm. This was clinically meaningful, meaning that malignant lesions were rarely omitted. In a previous study for 148 breast lesions, it was found that the sensitivity and specificity of SE by Tsukuba Score were 96.6% and 40.0% (cut-off value, Score ≥ 2), respectively, and our results were moderately better than this study. Nevertheless, although the SR part of this study focused on FLR as we did, its sensitivity of 90.9% was much higher than ours because the cut-off value was only 1.5. On the contrary, the specificity of 53.3% was much lower than ours (24). The other study, which included 164 breast lesions, showed that sensitivity and specificity were 39% and 94% for ES by Tsukuba Score (the cut-off point for malignancy was between TS 3 and TS 4 the same as the WFUMB guidelines) and 57% and 83% for SR, with a cut-off of 2.5 (25). At the same time, it is worth noticing that the specificity of SR was significantly higher in the cohort by size ≤20 mm than in the cohort by size >20 mm in our study. This indicated that different parameters could be selected for a more applicable application range. But, in another study for 113 solid breast lesions, measuring less than 3 cm, the ROC curve for ES by Tsukuba Score showed a sensitivity of 92% and specificity of 95% at the cut-off value of >3 (26). Such high diagnostic power might be related to sample selection. Strain elastography could undoubtedly help diagnose breast cancer, but related studies reflected subjectivity and large differences in cut-off value. The WFUMB recommendation criteria could effectively detect breast cancer and avoid missed diagnosis, which has high clinical application value.

Since the appearance of SWE, elasticity assessment methods have entered the quantitative era from qualitative and semi-quantitative methods. As far as the overall study was concerned, there was no definite evidence to indicate which was better, SE or SWE (18). However, the role of SWE in the diagnosis, differentiation, efficacy evaluation and prediction of breast cancer was obvious (13, 14, 27). One study demonstrated the superior predictive value of Emax in combination with BI-RADS category 4a in breast cancer screening (28). Another research found that Emax was the best parameter for classifying breast lesions, with a maximum AUC of 0.90 (95%CI: 0.77-1.00) (29). In our research, the diagnostic ability of Emax of SWE was not distinguished. Even with a cut-off of 60kPa, the sensitivity was still lower than that of ES. We thought the reason was the lack of marginal information supplements. In the 5 scores of ES, the range of high stiffness was larger than the identifiable area of the lesion, while the value of SWE fails to consider the edge information. ​Therefore, we revised it based on the WFUMB recommendation and considered choosing the parameter Emax-A’-S2.5mm to replace the parameter Emax of the original lesion by expanding the shell of the lesion. Under the same conditions (cut-off value: 80 kPa), the sensitivity was substantially increased, from 80.3% to 95.8%. In particular, the sensitivity was as high as 100% for lesions with sizes larger than 2 cm. In the study of 182 cases of breast solid lesions, the Emax of the shell of the lesion was the most valuable indicator of the elasticity of breast cancer diagnosis (30). In another study of breast non-mass lesions, Emax of the shell at 2.5 mm had better diagnostic efficiency than other parameters (31). In a few shell-related studies, although shell values were different, the diagnostic ability of SWE for breast cancer in the combined area of lesion and shell was significantly improved. In our study, the combined diagnostic capability was further improved by SE and SWE with shell(the ES≥4 and Emax-A’-S2.5mm>80kPa for the malignant lesions) in all lesions.

​The probability of malignant BI-RADS category 4 breast lesions ranged from 2% to 95%, while that of category 4a lesions was only 2% to 10%. A large number of benign lesions were biopsied, causing physical and psychological harm to the patient. Numerous studies have aimed to improve the accuracy of diagnosis by elastography to reduce the biopsy rate of benign lesions. ​Our previous study found that ultrasound and strain elastography could help optimize treatment recommendations for BI-RADS-MRI category 4a lesions (22). In a multicellular study, 104 (47.1%) of 221 false-positive results were correctly diagnosed as non-breast cancer by conventional ultrasound combined with SE (32). ​A study on solid breast masses found that SWE plus conventional ultrasound BI-RADS was more valuable in distinguishing benign and malignant breast lesions than color doppler or SWE alone (27). In our research, 27.4%-61.9% of category 4a lesions could be avoided biopsy by the combination of SE and SWE, which might contain no more than 2 cases of breast cancer smaller than 1cm, and would have the chance to be saved in the follow-up. Similar to our findings, in an international multicenter trial, reclassification of BI-RADS category 4a lesions with SWE and SE combined with routine ultrasound helped decrease the number of unnecessary biopsies in breast cancer diagnosis by approximately 35% while keeping the rate of undetected malignancy below 2% as defined by ACR BI-RADS 3 definition (15).

Our study had the following limitations. Firstly, this was a single-center confirmatory prospective study, and we would try to combine other centers for further exploration in the future. Secondly, because our results were obtained by only one instrument and did not compare the differences between machines, the generalization of the results was limited, but at the same time, the bias between instruments was avoided. Thirdly, we had more benign cases than malignant cases, which, although more in line with clinical reality, also contributed to the discrepancy between the results and other studies. Finally, our physicians were experienced in breast diagnostics, so we did not perform consistency verification, as the other study had shown that training could significantly improve consistency (33).

In conclusion, ​the clinical use of ultrasound elastography for breast lesions following WFUMB guidelines and recommendations had good application prospects in the real world. The elastography score for SE had high sensitivity, which was beneficial in the diagnosis of breast cancer. SWE could get similar results after our modification. The combination of SWE and SE could effectively reduce the biopsy rate of BI-RADS category 4a lesions.

## Data availability statement

The raw data supporting the conclusions of this article will be made available by the authors, without undue reservation.

## Ethics statement

The studies involving human participants were reviewed and approved by Ethics Committee of our hospital. The patients/participants provided their written informed consent to participate in this study.

## Author contributions

LT and YW have contributed equally to this work and share first authorship. LJ and MC contributed to project administration, conceptualization, supervision, and writing-editing. LT and YW contributed to writing-original drafts, data analysis, and formal analysis. LT, YW, and PC contributed to image acquisition, investigation, and validation. All authors contributed to the article and approved the submitted version.

## Funding

This study was supported by the National Natural Science Foundation of China (Grant no. 81470079), the Natural Science Foundation of Shanghai (Grant no. 18ZR1434800), and the Shanghai Municipal Health and Family Planning Commission (Grant no. 201940059).

## Acknowledgments

The author thanks everyone who participated in this study.

## Conflict of interest

The authors declare that the research was conducted in the absence of any commercial or financial relationships that could be construed as a potential conflict of interest.

The reviewer WZ declared a shared affiliation with the authors to the handling editor at the time of review.

## Publisher’s note

All claims expressed in this article are solely those of the authors and do not necessarily represent those of their affiliated organizations, or those of the publisher, the editors and the reviewers. Any product that may be evaluated in this article, or claim that may be made by its manufacturer, is not guaranteed or endorsed by the publisher.

## Abbreviations

SE, strain elastography; SWE, shear wave elastography; WFUMB, World Federation of Ultrasound in Medicine and Biology; BI-RADS, Breast Imaging Reporting and Data System; US, ultrasound; ES, elastography score; SR, strain ratio; AUC, area under the curve; CI, confidence interval; ACR, American College of Radiology.
