# Supplementary material for: Clinical use and adjustment of ultrasound elastography for breast lesions followed WFUMB guidelines and recommendations in the real world
Source: Front Oncol. 2022 Nov 24;12:1022917. doi: 10.3389/fonc.2022.1022917 (PMC9730323; doi:10.3389/fonc.2022.1022917)
Supplement: Supplementary file 1 [file Table_1.docx]

**Table The sensitivity, specificity, and the AUC for the 5 samples, the average, and all lesions**

|  | Cut-off value | Sensitivity | Specificity | AUC | 95%CI | |
| --- | --- | --- | --- | --- | --- | --- |
| Sample 1 | ES4-5 | 98.6% | 54.9% | 0.77 | 0.69 | 0.85 |
|  | SR>4.5 | 50.7% | 80.3% | 0.65 | 0.56 | 0.75 |
|  | Emax-A>80 kPa | 80.3% | 57.7% | 0.69 | 0.60 | 0.78 |
|  | Emax-A’-S2.5mm>80kPa | 95.8% | 38.0% | 0.67 | 0.58 | 0.76 |
| Sample 2 | ES4-5 | 98.6% | 50.7% | 0.75 | 0.66 | 0.83 |
|  | SR>4.5 | 50.7% | 81.7% | 0.66 | 0.57 | 0.75 |
|  | Emax-A>80 kPa | 80.3% | 53.5% | 0.67 | 0.58 | 0.76 |
|  | Emax-A’-S2.5mm>80kPa | 95.8% | 38.0% | 0.67 | 0.58 | 0.76 |
| Sample 3 | ES4-5 | 98.6% | 66.2% | 0.82 | 0.75 | 0.90 |
|  | SR>4.5 | 50.7% | 84.5% | 0.68 | 0.59 | 0.77 |
|  | Emax-A>80 kPa | 80.3% | 74.6% | 0.77 | 0.70 | 0.85 |
|  | Emax-A’-S2.5mm>80kPa | 95.8% | 49.3% | 0.73 | 0.64 | 0.81 |
| Sample 4 | ES4-5 | 98.6% | 59.2% | 0.79 | 0.71 | 0.87 |
|  | SR>4.5 | 50.7% | 80.3% | 0.65 | 0.56 | 0.75 |
|  | Emax-A>80 kPa | 80.3% | 62.0% | 0.71 | 0.62 | 0.80 |
|  | Emax-A’-S2.5mm>80kPa | 95.8% | 43.7% | 0.70 | 0.61 | 0.78 |
| Sample 5 | ES4-5 | 98.6% | 66.2% | 0.82 | 0.75 | 0.90 |
|  | SR>4.5 | 50.7% | 85.9% | 0.68 | 0.59 | 0.77 |
|  | Emax-A>80 kPa | 80.3% | 60.6% | 0.70 | 0.62 | 0.79 |
|  | Emax-A’-S2.5mm>80kPa | 95.8% | 40.8% | 0.68 | 0.59 | 0.77 |
| Average | ES4-5 | 98.6% | 59.4% | 0.79 | 0.71 | 0.87 |
|  | SR>4.5 | 50.7% | 82.5% | 0.67 | 0.58 | 0.76 |
|  | Emax-A>80 kPa | 80.3% | 61.7% | 0.71 | 0.62 | 0.80 |
|  | Emax-A’-S2.5mm>80kPa | 95.8% | 42.0% | 0.69 | 0.60 | 0.78 |
| All lesions | ES4-5 | 98.6% | 59.1% | 0.79 | 0.74 | 0.84 |
|  | SR>4.5 | 50.7% | 81.8% | 0.66 | 0.59 | 0.74 |
|  | Emax-A>80 kPa | 80.3% | 63.5% | 0.72 | 0.65 | 0.79 |
|  | Emax-A’-S2.5mm>80kPa | 95.8% | 43.3% | 0.70 | 0.63 | 0.76 |

AUC, area under the characteristic curve; CI, confidence interval.
